# Ethnic-based separation in maternity Departments in Israel – a balanced practical view

**DOI:** 10.1186/s13584-018-0262-8

**Published:** 2018-11-21

**Authors:** Jonathan Halevy

**Affiliations:** Shaare Zedek Medical Center, Jerusalem, Israel

## Abstract

Ethnic-based separation in public hospitals in Israel is a sensitive issue that was recently brought forward by the media and was recently discussed in the Israel Journal of Health Policy Research.

The above paper maintains that ethnic separation in inpatient rooms does take place some of the time and this runs contrary to the ethos of neutrality in medicine. The authors recommend implementing a national policy that prohibits ethnic-based separation in hospital inpatient rooms.

In this commentary I point to the fact that the authors’ research indicates that often times ethnic separation is not based on racism, and while the call for unequivocal prohibition of discriminatory ethnic-based separation is of course morally justified, such an across-the-board prohibition is actually an imposition of mixed rooms under all circumstances.

I recommend a more balanced and still ethically acceptable approach: any request by patients for a separate room that is overtly based on ethnic discrimination should be immediately rejected and that hospital directors should be called upon by the Ministry of Health not to take a back seat on this issue, to be proactive in explaining to the staff the importance of absolute avoidance of any discriminatory considerations in the placement of patients, and to monitor the extent of ethnic separation expecting to see in every department ethnically mixed rooms.

## Commentary

In a recent IJHPR article, Keshet and Popper-Giveon discuss ethnic-based separation in public hospitals in Israel [1]. This is a sensitive issue that was recently brought forward by the media, accusing directors of hospitals and physicians and nurses running maternity wards in public hospitals in Israel with racism as they separate patients into different rooms based on their ethnic origin.

The authors conclude, based on a mixed methodology using quantitative (survey of a representative sample of the Israeli population) and qualitative (50 in-depth interviews with nurses, physicians, and managers in 11 public hospitals) tools that ethnic separation in inpatient rooms does take place some of the time and this runs contrary to the ethos of neutrality in medicine. The authors go on to recommend implementation of a specific national policy that prohibits ethnic-based separation in hospital inpatient rooms so that segregation does not become institutionalized.

It is no secret that racism exists in most societies and one cannot rule out the possibility that segregation of Jewish and Arab patients in different rooms in maternity departments in Israel is at least sometimes, unfortunately, due to ethnic discrimination.

Does Keshet and Popper-Giveon’s paper prove the existence of ethnic discrimination in Israel’s maternity departments and quantify its magnitude? Moreover, is their call for a national policy that prohibits ethnic-based separation in hospital’s inpatient rooms the right solution to the problem?

According to the authors, whose paper discusses the situation only in maternity departments, there could be three reasons for nurses placing new mothers in the departments’ rooms to put Jewish and Arab patients in separate rooms: 1) to promote cultural compatibility between patients, 2) to avoid unnecessary confrontation, and 3)ethnic discrimination, related to racism.

The quantitative part of their research, the survey, showed that 30% of Jews and 21% of Arabs agree that patients should be allowed to choose to be placed in an inpatient room in which only patients of their own ethnic group are hospitalized. The in-depth interview revealed evidence of demands for ethnic separation made at times by Jewish patients, which are sometimes met by the nurses. In addition, some nurses reported having separated Jewish and Arab patients of their own accord.

This paper is important in raising awareness in the medical community of a pattern of behavior of medical staff that should be condemned and eliminated. However, its main limitation is that it cannot assess the magnitude of the problem. Moreover, the authors’ call for a national policy that prohibits discriminatory ethnic-based separation in hospital inpatient rooms, while justified in principle, is not practical.

The authors named the three reasons listed above that motivate nurses to separate Jewish and Arab patients. Two of these reasons stem from practical considerations (promoting cultural compatibility between patients and avoiding unnecessary tension and confrontation) that cannot be condemned. If a Jewish secular patient who is female asks to preferably be placed in a room with secular women so that desecration of the Sabbath rules by herself will not disturb the neighbor in the room and the nurse responds positively – should that be considered discriminatory ethnic separation? If an ultra-orthodox Jewish patient asks the nurse to preferably be placed in a room with ultra-orthodox mothers so she could speak Yiddish to them, Yiddish being their mother language, and they could accommodate their large respective families and not disturb mothers who have smaller families; should this be considered discriminatory ethnic-based separation? And the same applies to an ultra-religious Moslem mother.

The call for unequivocal prohibition of discriminatory ethnic-based separation is of course morally justified but how is it going to be implemented in view of the examples listed above and similar other scenarios? Are the authors calling for the imposition of mixed rooms under all circumstances?

A more balanced and still ethically acceptable recommendation would be to refuse any request of patients that is not based on the types of considerations described above or that is overtly based on ethnic discrimination. The practical way for managers to verify that such a balanced recommendation is being implemented is by verifying that in the maternity departments a significant proportion of the rooms are ethnically mixed even if there are also rooms in which all the patients are from the same culture or ethnic group. I believe it is the duty of the managers to verify this fact.

I do agree with the authors that the expressions of denial from heads of hospitals regarding the existence of any ethnic-based separation in their institutions reflects a tendency to ignore the problem for fear of bad media. The hospital directors should be called upon by the Ministry of Health not to take a back seat on this issue, and to be proactive in meeting with the physicians and nurses of their maternity departments, to explain the importance of absolute avoidance of any discriminatory ethnic considerations in the placement of patients.

A periodic survey of the occupants of rooms in the department can be used to monitor the extent of ethnic separation and serve to limit any tendencies toward discriminatory separation.

## Conclusions

This commentary deals with ethnic-based separation in public hospitals in Israel. I suggest a balanced, practical, and morally acceptable approach.
